# Supplementary material for: Transcriptome Analysis of Co-Cultures of THP-1 Human Macrophages with Inactivated Germinated Trichophyton rubrum Conidia
Source: J Fungi (Basel). 2023 May 12;9(5):563. doi: 10.3390/jof9050563 (PMC10219549; doi:10.3390/jof9050563)
Supplement: Supplementary file 1 [file jof-09-00563-s001.zip › jof-2312914-supplementary.pdf]

Supplementary Table S1. Primers used for qPCR analysis.

ID	Gene Product Name	Primer sequence (5′- 3′)	Fragment	Reference
**CRLF2**<br>NM_001012288.2	cytokine receptor like factor 2	F: TGAGATTTTCGTGGCATCAG<br>R: TTCTATGGTGACGTTGCAGG	147 bp	This paper
**MMP10**<br>NM_002425.3	matrix metallopeptidase 10	F:GGAGTTGAGCCTAAGGTTGATG<br>R: TCGCCTAGCAATGTAACCAG	145 bp	This paper
**ANKRD1**<br>NM_014391.2	ankyrin repeat domain 1	F:GGTGAGACTGAACCGCTATAAG<br>R: GGCTGTCGAATATTGCTTTGG	140 bp	This paper
**CCL24**<br>NM_002991.2	C-C motif chemokine ligand 24	F: TTCTGTTCCTTGGTGTCTGTG<br>R: CACTCGGTTCTCAGGAATTCTC	109 bp	This paper
**SLC43A2**<br>NM_001284498.1	solute carrier family 43 member 2	F: GGGCTTTTACTCCTACCTGTG<br>R: AATTTAGCATCTCGTCCTGGG	140 bp	This paper
**CSF1**<br>NM_000757.6	colony stimulating factor 1	F: CGCTTCAGAGATAACACCCC<br>R: TCATAGAAAGTTCGGACGCAG	128 bp	This paper
**CXCL1**<br>NM_001511.3	chemokine (C-X-C motif) ligand 1 (melanoma growth stimulating activity, alpha)	F: AACCGAAGTCATAGCCACAC<br>R: GTTGGATTTGTCACTGTTCAGC	109 bp	This paper
**CXCL2**<br>NM_002089.4	chemokine (C-X-C motif) ligand 2	F: AACCGAAGTCATAGCCACAC<br>R: TCTGGTCAGTTGGATTTGCC	116 bp	This paper
**CXCL3**<br>NM_002090.2	chemokine (C-X-C motif) ligand 3	F: AACCGAAGTCATAGCCACAC<br>R: GTGCTCCCCTTGTTCAGTATC	105 bp	This paper
**CXCL8**	chemokine (C-X-C motif) ligand 8	F: TCCTGATTTCTGCAGCTCTG	138 bp	This paper

NM_000584.4		R: GTCCACTCTCAATCACTCTCAG		
**IL-32**<br>NM_001012631.2	interleukin 32	F: AAACCTGCTTATACTTCCCTGG<br>R:CTTTATACATCACCCAGTCTCAGG	138 bp	This paper
**S1PR1**<br>NM_001400.4	sphingosine-1-phosphate receptor 1	F:GGGAAGGGAGTATGTTTGTGG<br>R:AGGAAGAGGCGGAAGTTATTG	127 bp	This paper
**TLR8**<br>NM_016610.3	Member of the Toll-like receptor family (TLR)	F: TGGTTCCTGTGAGTTATGCG<br>R: CACATATTTGCCCACCGTTTG	139 bp	This paper
**TLR7**<br>NM_016562.4	toll-like receptor 7	F: TTCCTAAAACTCTGCCCTGTG<br>R: TGTGGTTAATGGTGAGGGTG	141 bp	This paper
**CD1D**<br>NM_001766.3	CD1d molecule	F: TCATTGTGGGCTTTACCTCC<br>R: TTCAATTCCTGAGCAGACCAG	148 bp	This paper
**FCGBP**<br>NM_003890.2	Fc fragment of IgG binding protein	F:GTGACGTTGACTCCAAACAAG<br>R: ACGGACACAGATGCTAACTTC	83 bp	This paper
**ACTB**	Beta-actin	F:GTTGCGTTACACCCTTTCTTG<br>R:TGCTGTCACCTTCACCGTTC	154 bp	(DAI, Z. J. *et al.*, 2012)
**GAPDH**	Glyceraldehyde-3-phosphate dehydrogenase	F:AATCCCATCACCATCTTCCAG<br>R:GAGCCCCAGCCTTCTCCAT	118 bp	(MA *et al.*, 2015)

Supplementary Table S2. General features of RNA-seq reads mapped to the human reference genome (hg38).

Sample	Raw reads	High-quality reads	Mapped reads STAR	Total mapped reads (%)
THP1-7 (Control1)	92,503,239	88,111,148	83,912,255	95.23
THP1-8 (Control2)	93,650,266	88,951,289	84,891,979	95.44
THP1-9 (Control3)	102,973,651	98,294,201	93,696,535	95.32
Co-THP1-10 (Treatment1)	93,481,073	89,058,976	83,691,093	93.97
Co-THP1-11 (Treatment2)	95,946,772	91,427,461	86,957,411	95.11
Co-THP1-12 (Treatment3)	98,841,212	93,565,192	88,590,607	94.68

Supplementary Table S3. RNA-seq genes from the co-culture of THP-1 macrophages with *T.rubrum* CGI.
($P < 0.05$) up regulated → log2 (Fold Change) ≥ 1.0 and down-regulated → log2 (Fold Change) ≤ −1.0.
DEGs = 83 genes

		Co-THP1 vs. THP1
**ID**	**Log2FoldChange**	**Gene Product Name**
CRLF2	2.79	cytokine receptor like factor 2
GREM1	2.75	gremlin 1, DAN famIy BMP antagonist
EBF1	2.59	EBF transcription factor 1
MMP10	2.41	matrix metallopeptidase 10
ANKRD1	2.21	ankyrin repeat domain 1
LIF	2.14	LIF interleukin 6 family cytokine
HIVEP2	2.12	human immunodeficiency virus type I enhancer binding protein 2
CCL24	1.92	C-C motif chemokine ligand 24
SLC43A2	1.92	solute carrier family 43 member 2
CSF1	1.90	colony stimulating factor 1
CXCL3	1.88	C-X-C motif chemokine ligand 3
TFPI2	1.88	tissue factor pathway inhibitor 2
GRASP	1.86	general receptor for phosphoinositides 1 associated scaffold protein
NR4A3	1.81	nuclear receptor subfamily 4 group A member 3
ATP1B4	1.78	ATPase Na+/K+ transporting family member beta 4

PRSS22	1.77	serine protease 22
COL12A1	1.75	collagen type XII alpha 1 chain
CXCL1	1.72	C-X-C motif chemokine ligand 1
CXCL8	1.71	C-X-C motif chemokine ligand 8
MGLL	1.68	monoglyceride lipase
MGAM	1.67	maltase-glucoamylase
IFI44L	1.57	interferon induced protein 44 like
FMN1	1.51	formin 1
BAALC-AS2	1.49	BAALC antisense RNA 2
TMEM119	1.43	transmembrane protein 119
FAM216B	1.41	family with sequence similarity 216 member B
SLC28A3	1.40	solute carrier family 28 member 3
NRG1	1.37	neuregulin 1
P3H2	1.37	prolyl 3-hydroxylase 2
SCG5	1.36	secretogranin V
CLEC5A	1.36	C-type lectin domain containing 5A
TNFAIP6	1.35	TNF alpha induced protein 6
MMP8	1.35	matrix metallopeptidase 8
BIRC3	1.33	baculoviral IAP repeat containing 3
TNFRSF18	1.30	TNF receptor superfamily member 18
MME	1.30	membrane metalloendopeptidase
SERPINE1	1.29	serpin family E member 1
IFIT1	1.28	interferon induced protein with tetratricopeptide repeats 1
SYN2	1.28	synapsin II
(40)IL32	1.27	interleukin 32
TIMP4	1.27	TIMP metallopeptidase inhibitor 4
KIAA1644	1.25	[]
PHF24	1.23	PHD finger protein 24

MET	1.23	MET proto-oncogene, receptor tyrosine kinase
SRC	1.21	SRC proto-oncogene, non-receptor tyrosine kinase
LOC653513	1.19	[]
HR	1.18	HR lysine demethylase and nuclear receptor corepressor
CLDN1	1.17	claudin 1
FCAR	1.17	Fc fragment of IgA receptor
IGFN1	1.16	immunoglobulin-like and fibronectin type III domain containing 1
TNF	1.16	tumor necrosis factor
TGFB3	1.16	transforming growth factor beta 3
TDRD6	1.15	tudor domain containing 6
LINC00877	1.15	long intergenic non-protein coding RNA 877
SEMA3C	1.14	semaphorin 3C
CXCL2	1.10	C-X-C motif chemokine ligand 2
NCF1	1.09	neutrophil cytosolic factor 1
MX1	1.06	MX dynamin like GTPase 1
STC1	1.05	stanniocalcin 1
MMP9	1.05	matrix metallopeptidase 9
TRAF1	1.05	TNF receptor associated factor 1
RRAD	1.05	RRAD, Ras related glycolysis inhibitor and calcium channel regulator
SOD2	1.04	superoxide dismutase 2
LINC01050	1.02	long intergenic non-protein coding RNA 1050
TBX21	1.00	T-box 21
CD1D	-1.03	CD1d molecule
PLK2	-1.04	polo like kinase 2
TLR7	-1.04	toll like receptor 7
ROBO4	-1.05	roundabout guidance receptor 4
TLR8	-1.05	toll like receptor 8
C2orf91	-1.08	chromosome 2 open reading frame 91

VCAN	-1.11	versican
SH3TC2	-1.13	SH3 domain and tetratricopeptide repeats 2
MMRN2	-1.19	multimerin 2
PDE7B	-1.20	phosphodiesterase 7B
FCGBP	-1.22	Fc fragment of IgG binding protein
HPSE	-1.24	heparanase
NLRP12	-1.26	NLR family pyrin domain containing 12
LINC01537	-1.31	long intergenic non-protein coding RNA 1537
BBOX1	-1.44	gamma-butyrobetaine hydroxylase 1
NTS	-1.67	neurotensin
S1PR1	-1.69	sphingosine-1-phosphate receptor 1
P2RY12	-1.88	purinergic receptor P2Y12

Gene expression values are expressed in log_2 fold change.
